# Urinary metabolomics of young Italian autistic children supports abnormal tryptophan and purine metabolism

**DOI:** 10.1186/s13229-016-0109-5

**Published:** 2016-11-24

**Authors:** Federica Gevi, Lello Zolla, Stefano Gabriele, Antonio M. Persico

**Affiliations:** 1 Department of Ecological and Biological Sciences, University of Tuscia, Viterbo, Italy; 2 Unit of Child and Adolescent Neuropsychiatry, Laboratory of Molecular Psychiatry and Neurogenetics, University Campus Bio-Medico, Rome, Italy; 3 Unit of Child and Adolescent Neuropsychiatry, Interdepartmental Program “Autism 0-90”, “Gaetano Martino” University Hospital, University of Messina, Messina, Italy; 4 Mafalda Luce Center for Pervasive Developmental Disorders, Milan, Italy

**Keywords:** Autism, Autism spectrum disorder, Kynurenine, Melatonin, Metabolomics, Purinergic signaling, Quinolinic acid, Serotonin, Tryptophan

## Abstract

**Background:**

Autism spectrum disorder (ASD) is still diagnosed through behavioral observation, due to a lack of laboratory biomarkers, which could greatly aid clinicians in providing earlier and more reliable diagnoses. Metabolomics on human biofluids provides a sensitive tool to identify metabolite profiles potentially usable as biomarkers for ASD. Initial metabolomic studies, analyzing urines and plasma of ASD and control individuals, suggested that autistic patients may share some metabolic abnormalities, despite several inconsistencies stemming from differences in technology, ethnicity, age range, and definition of “control” status.

**Methods:**

ASD-specific urinary metabolomic patterns were explored at an early age in 30 ASD children and 30 matched controls (age range 2–7, M:F = 22:8) using hydrophilic interaction chromatography (HILIC)-UHPLC and mass spectrometry, a highly sensitive, accurate, and unbiased approach. Metabolites were then subjected to multivariate statistical analysis and grouped by metabolic pathway.

**Results:**

Urinary metabolites displaying the largest differences between young ASD and control children belonged to the tryptophan and purine metabolic pathways. Also, vitamin B_6_, riboflavin, phenylalanine-tyrosine-tryptophan biosynthesis, pantothenate and CoA, and pyrimidine metabolism differed significantly. ASD children preferentially transform tryptophan into xanthurenic acid and quinolinic acid (two catabolites of the kynurenine pathway), at the expense of kynurenic acid and especially of melatonin. Also, the gut microbiome contributes to altered tryptophan metabolism, yielding increased levels of indolyl 3-acetic acid and indolyl lactate.

**Conclusions:**

The metabolic pathways most distinctive of young Italian autistic children largely overlap with those found in rodent models of ASD following maternal immune activation or genetic manipulations. These results are consistent with the proposal of a purine-driven cell danger response, accompanied by overproduction of epileptogenic and excitotoxic quinolinic acid, large reductions in melatonin synthesis, and gut dysbiosis. These metabolic abnormalities could underlie several comorbidities frequently associated to ASD, such as seizures, sleep disorders, and gastrointestinal symptoms, and could contribute to autism severity. Their diagnostic sensitivity, disease-specificity, and interethnic variability will merit further investigation.

**Electronic supplementary material:**

The online version of this article (doi:10.1186/s13229-016-0109-5) contains supplementary material, which is available to authorized users.

## Background

Autism spectrum disorder (ASD) represents a highly heterogeneous collection of neurodevelopmental conditions characterized by social and communication deficits, stereotypic and rigid patterns of behavior, restricted interests, and unusual sensory processing with onset in early childhood [1]. The prevalence of autism has increased significantly during the last two decades from 2–5/10,000 to 1:68 children [2, 3]. Changes in diagnostic criteria and increased attention by the medical community have certainly contributed to this trend [4]. Also, increasing parental age at conception has been shown to confer ASD risk [5], as well as some environmental factors, active especially during critical periods in prenatal/early postnatal neurodevelopment [6]. Finally, genetic susceptibility plays a prominent role in ASD pathogenesis through complex and heterogeneous underpinnings, ranging from rare variants endowed with full penetrance to common variants each explaining very small proportions of the overall phenotypic variance, either alone or through gene × environment interactions [7, 8].

Despite major advances in our understanding of the pathophysiology of ASD, this level of complexity and interindividual heterogeneity has largely hampered the translation of scientific knowledge into more effective clinical practices. ASD is still diagnosed exclusively through observation, standardized behavioral scales, and parental interviews; developmental trajectories of ASD children are periodically monitored but cannot be reliably predicted especially at an early age. Sensitive and specific quantitative biomarkers, measurable through laboratory, brain imaging, and/or electrophysiological techniques, could greatly aid clinicians in providing earlier diagnoses, more timely referrals to behavioral intervention programs, and evidence-based prognostic predictions [9].

Metabolomic technologies offer a sensitive means to search human biofluids for metabolite profiles potentially usable as biomarkers for neurodevelopmental disorders. A few studies have recently begun exploring the potential of urinary metabolomics in identifying ASD-specific metabolic patterns or in stratifying ASD patients into pathophysiologically meaningful subgroups [10–17]. Most studies have been performed on urines [10–16]; one study has explored blood plasma [17]. The analytical platforms most commonly used to identify and quantify metabolites are gas or liquid chromatography combined with mass spectroscopy (gas chromatography (GC)-mass spectroscopy (MS) and liquid chromatography (LC)-MS, respectively) [12, 16] and nuclear magnetic resonance spectroscopy (NMR) [10, 13, 14, 16, 18, 19]. In general, NMR displays greater speed and good reproducibility but also lower sensitivity compared to MS. Hence, MS- and NMR-based techniques should be viewed as complementary, not as superimposable approaches. An initial study, using 1H-NMR methods, showed an abnormal composition of urinary solutes indicative of perturbations in (a) the tryptophan/nicotinic acid metabolic pathway, (b) sulfur and amino acid metabolisms, and (c) gut microbiome, with an excess of several gut-derived co-metabolites [10]. Two other studies presumably assessing the same clinical sample with two different NMR-based technologies largely replicated these initial findings [13, 14]. Other studies using GC-MS, either alone [12, 15] or in combination with liquid chromatography [11], also identified perturbations in amino acid metabolism and gut microbial co-metabolites, as well as metabolic signatures of oxidative stress. Only one very recent study used both NMR and LC-MS, providing support for abnormalities in tryptophan metabolism, gut bacterial-derived compounds, purine and pyrimidine metabolism [16]. The only study exploring blood plasma reported metabolomic patterns compatible with (a) mitochondrial dysfunction, yielding reduced energy production and unbalanced redox status, (b) excess gut microbial co-metabolites, and (c) unbalances in various metabolic pathways, such as the Krebs cycle [17]. Collectively, metabolomic studies performed to this date suggest that autistic patients may share several metabolic abnormalities, especially involving some amino acid metabolisms, energy production, and oxidative stress, as well as the gut microbiome.

Moving from broad metabolic pathways to single compounds unveils inconsistencies between studies, which may stem from several potential confounds. Interethnic differences in the gut microbiota, stemming from differences in the nutrient composition of local diets, as well as age-related changes in both gut microbiota and human metabolism indeed require that case and control samples be tightly matched for these two variables. Age-related changes may be especially relevant to studies of ASD, where we have recently reported levels of urinary *p*-cresol to be elevated in autistic children compared to age-matched controls both in Italy and in France, but exclusively up until 8 years of age [20, 21]. Similar age-related changes in ASD have been previously described for other parameters, such as brain serotonin synthesis capacity [22, 23] and excessive head growth rates [24]. Finally, some studies have contrasted ASD patients with unrelated population controls [11, 14, 16, 17], while others have enrolled unaffected siblings as controls [15] and one study has used both [10]. These strategies are not equivalent, as first-degree relatives often fall within the broad autism spectrum (i.e., they display behavioral phenotypes intermediate between patients and population controls) [25]. In addition, siblings may carry protective gene variants with peculiar functional correlates, possibly distinct from the metabolic patterns of unrelated typically developing children.

Taking into consideration these methodological issues, in order to maximize the probability of reliably detecting differences in urinary metabolic patterns, we focused on autistic and unrelated typically developing children 2–8 years old, tightly matched by age, sex, Italian ancestry, and city of origin within the country [20]. To ensure broad metabolite detection coverage on urine samples, which comprise molecules generated both by human cells and by the gut microbiome, we employed hydrophilic interaction chromatography (HILIC)-LC-electrospray ionization (ESI)-MS, a technology particularly suitable to separate simple and complex mixtures of carbohydrates, amino acids, glycosides, and other natural polar products in biological fluids, such as human urine and plasma [26, 27]. Applying this experimental approach, urinary metabolites most significantly distinguishing autistic from typically developing children were found to primarily fall into the tryptophan and purine metabolic pathways.

## Methods

### Subjects

Thirty children with idiopathic ASD and thirty typically developing controls were recruited in Central and Northern Italy. These represent the vast majority of the 64 cases and controls aged 3–7 years assessed for urinary *p*-cresol in our previous study [20]. Their demographic and clinical characteristics are summarized in Additional file 1: Table S1. Diagnostic assessments and medical screening have been previously described [20] (also see Additional file 2 with Supplementary Methods). Tight sex- and age-matching (±1 year) was applied to recruit typically developing children devoid of any overt ASD symptomatology among the offspring of clinical/academic personnel [20]. Mean age (±SEM) of cases and controls was 4.83 ± 0.30 and 5.03 ± 0.32 years, respectively (Student’s *t* = −0.459, 58 *df*, *P* = 0.648, n.s.), and the M:F ratio was 22:8. All cases and controls were of Italian descent and matched by geographical area or city of origin.

### Urine collection and metabolite extraction

First-morning urines were collected at home by parents using sterile containers untreated with preservatives and were brought to each clinical center the same morning in wet ice. Urine samples were then frozen, shipped in dry ice, and stored at −80 °C continuously until analysis.

Urinary specific gravity was measured by refractometry following centrifugation at 13,000*g* for 10 min) using a digital refractometer (Euromex Clinical Digital Refractometer RD.5712, NL) previously calibrated with LC-MS grade water.

Urine aliquots (200 μl) were mixed with 200 μl of methanol:acetonitrile:water (50:30:20), vortexed for 30 min at max speed at 4 °C and then centrifuged at 16,000*g* for 15 min at 4 °C. Supernatants were collected for metabolomic analysis. Quality controls (QCs) were obtained from a pooled mixture of 10 μl aliquots of all urine samples and were analyzed every 15 samples.

### HILIC-UHPLC

Metabolite separation was performed as previously described [28], by hydrophilic interaction chromatography (HILIC) using the Ultimate 3000 Rapid Resolution HPLC system (Dionex, Sunnyvale, CA), featuring a binary pump and vacuum degasser, well-plate autosampler with a six-port micro-switching valve, and a thermostated column compartment. A Phenomenex Luna 3 μm HILIC 200 A (150 × 2.0 mm) column, protected by a HILIC 4 × 2.0 mm ID guard column (Phenomenex, Torrance, CA), was used to perform metabolite separation over a phase B-to-phase A gradient lasting 35 min. For the HILIC separation, mobile phase “A” consisted in 50 mM ammonium acetate mixed with acetonitrile (95:5, *v*/*v*), while eluent “B” was composed of a mixture of 50 mM ammonium acetate:water plus acetonitrile (95:5, *v*/*v*). Acetonitrile, formic acid, and HPLC-grade water were purchased from Sigma-Aldrich (St. Louis, MO).

### Mass spectrometry

MS analysis was carried out on an electrospray hybrid quadrupole time-of-flight instrument MicroTOF-Q (Bruker-Daltonik, Bremen, Germany) equipped with an ESI ion source, as previously described [29]. Mass spectra for metabolite-extracted samples were acquired both in positive and in negative ion modes; only data produced in negative mode are shown, because more powerful in analyzing urinary samples. ESI capillary voltage was set at 4500 V (−) ion mode. The liquid nebulizer was set at 27 psi, and the nitrogen drying gas was set to a flow rate of 6 L/min. Dry gas temperature was maintained at 200 °C. Data were stored in centroid mode and acquired with a stored mass range of 50–1200 *m*/*z*. Instrument calibration was performed externally every day with 10 mM sodium hydroxide in 50% isopropanol: water, 0.1% formic acid. Automated internal mass scale calibration was performed through direct automated injection of the calibration solution at the beginning and at the end of each run by a six-port divert valve.

### Data elaboration and statistical analysis

Data were normalized by urinary specific gravity, because creatinine excretion may be abnormally reduced in ASD children [30]. Replicates were exported as mzXML files and processed through MAVEN.52 (available at http://genomics-pubs.princeton.edu/mzroll/index.php?show=index) [31]. Mass spectrometry chromatograms were elaborated for peak alignment, matching and comparison of parent and fragment ions, and tentative metabolite identification (within a 10-ppm mass deviation range between observed and expected results against the imported Kyoto Encyclopedia of Genes and Genomes (KEGG) database). Representative examples of mass determination and MS/MS fragmentation graphs are presented for kynurenine, melatonin, and tryptophan in Additional file 3: Figure S1. Multivariate statistical analyses were performed on the entire metabolomics data set using the MetaboAnalyst 3.0 software (http://www.metaboanalyst.ca) [32], which also overviewed data variance structure in an unsupervised manner and produced scatter plots.

Orthogonal partial least squares discriminant analysis (OPLS-DA), which defines a predictive model that describes the direction of the maximum covariance between a dataset (*X*) and class membership (*Y*), was then used to maximize the difference in metabolic profiles between cases and controls [33, 34]. OPLS-DA was performed using the Excel add-in Multibase package (Numerical Dynamics, Japan; http://www.numericaldynamics.com/) by applying orthogonal signal correction on the metabolite concentrations shifted, log_10_ transformed, centered, and scaled to unit variance.

Performance of the optimal model was tested by a receiver operating characteristic (ROC) curve analysis and the validation data set, as performed using MetaboAnalyst 3.0 software (http://www.metaboanalyst.ca) [32].

For case-control contrasts of single urinary metabolites, significance threshold was held at a nominal *P* < 0.05 with no correction for multiple testing, because (a) differences in single metabolite concentrations were tested only following significant differences in pathway enrichment were detected, (b) intra-pathway variability of single metabolites is non-independent, and (c) also different metabolic pathways are not fully independent, as some metabolites fall into more than one pathway. Detailed and summary statistics are provided in Additional files 4 and 5.

## Results

**Fig. 1:**
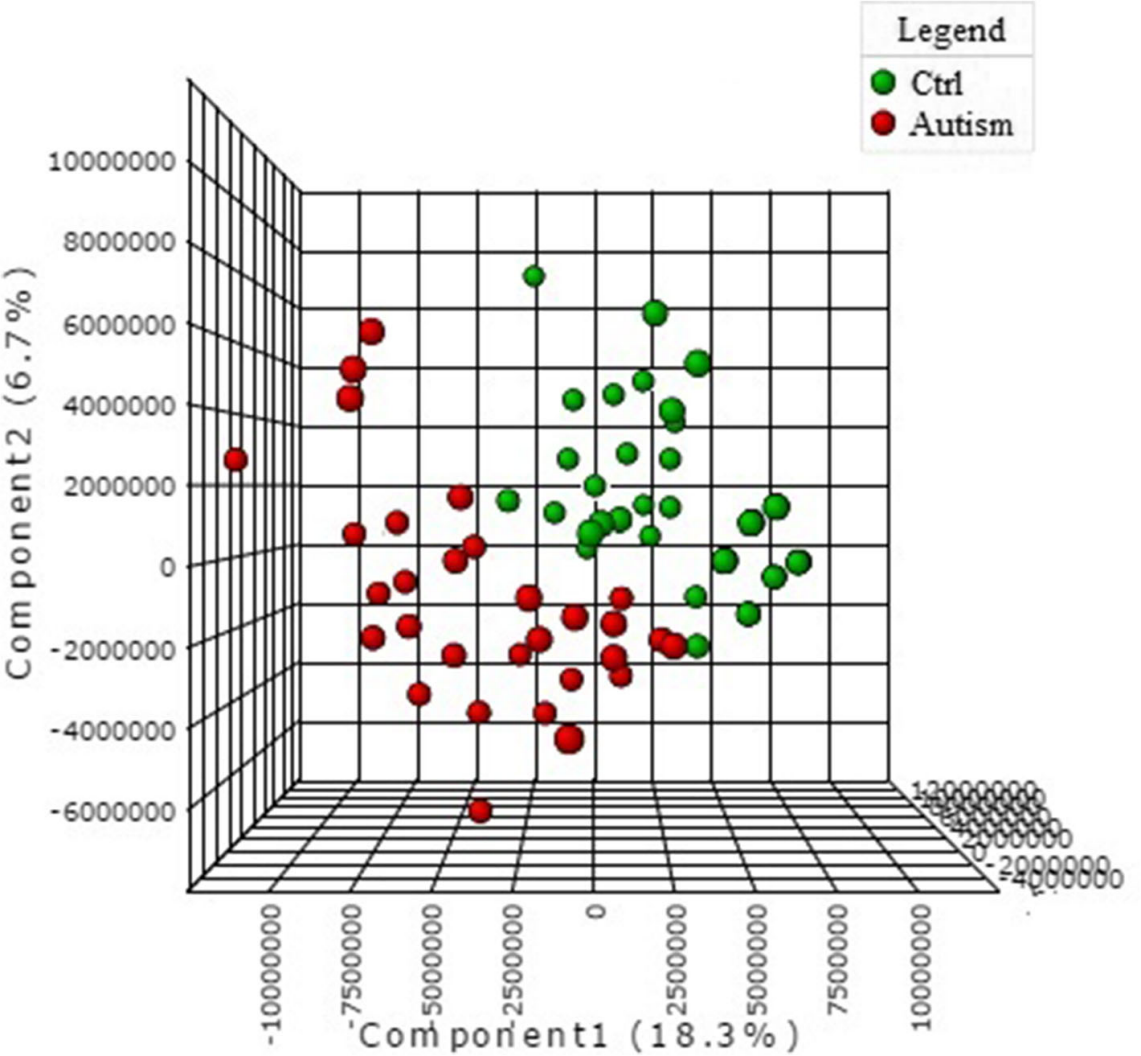
OPLS-DA 3D plot based on normalized and mean-centered data. Each data point represents the metabolome of a single individual. Some data points may be superimposed to each other

**Fig. 2:**
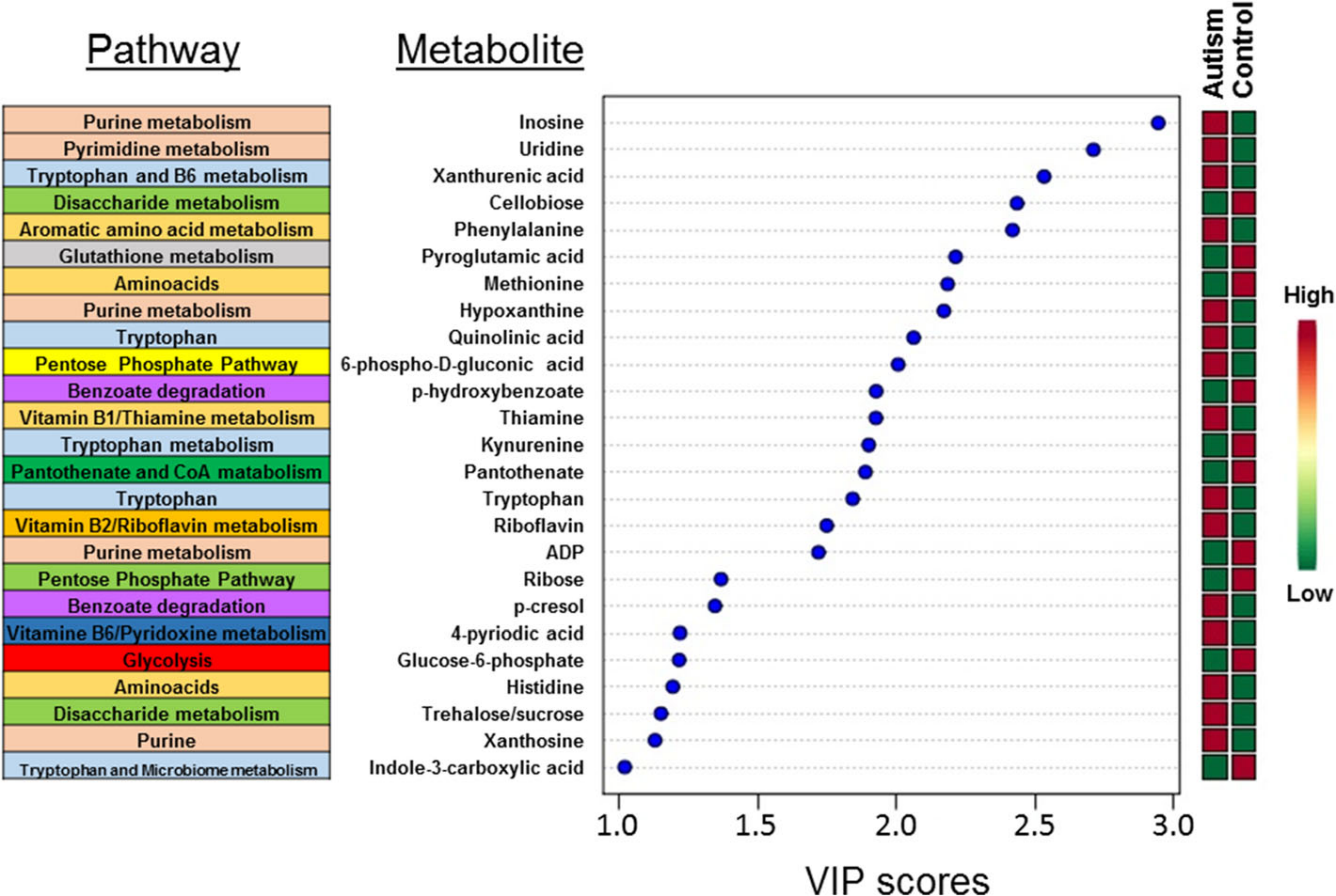
The top 25 most discriminating metabolite ASD cases from controls, ranked by variable importance in projection (VIP) scores, and their KEGG biochemical pathway. VIP scores >1.0 were considered significant

**Fig. 3:**
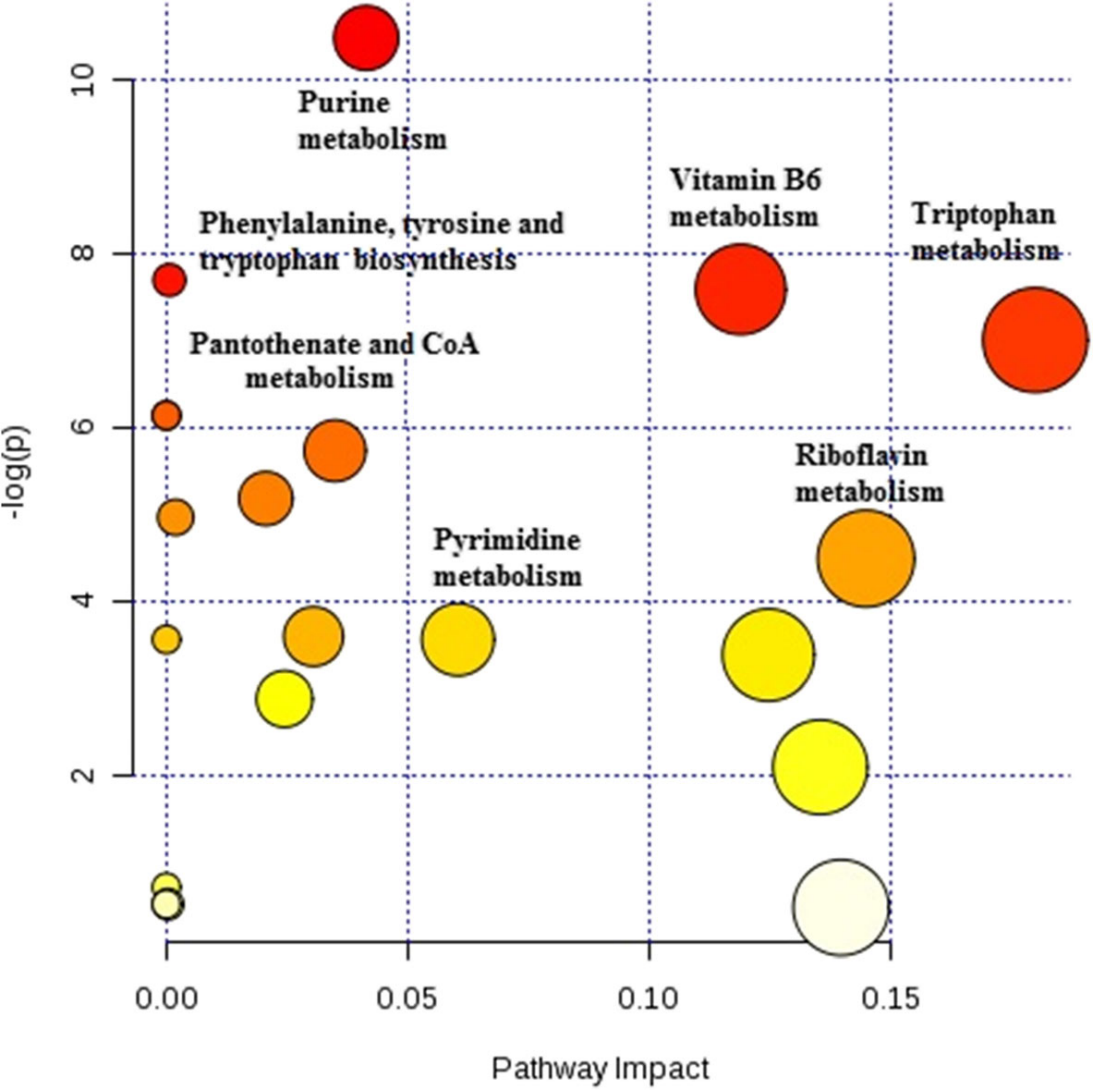
Metabolic pathway analysis plot. Color intensity (*white* to *red*) reflects increasing statistical significance, while *circle diameter* covaries with pathway impact. The graph was obtained plotting on the *y-axis* the −log of *p* values from the pathway enrichment analysis and on the *x-axis* the pathway impact values derived from the pathway topology analysis

The urinary metabolomes of young autistic and typically developing children are largely distinguishable on the three-dimensional OPLS-DA plot depicting the first three principal components (PC), which together explain 31.4% of the total variance (Fig. 1; accuracy, Q2 and R2 data are shown in Additional file 6). Approximately 10,000 peaks per sample were obtained referring to the KEGG database; among them, 202 metabolites were analyzed more precisely and identified. The top 25 most discriminating metabolites between cases and controls were further defined based on “variable influence on the projection” (VIP) scores >1 (Fig. 2). ROC analysis using this set of 25 metabolites yielded an AUC = 0.893 (95% CI 0.72–0.96), as shown in Additional file 7. The “metabolome overview” obtained through metabolic pathway analysis (MetPA) shows tryptophan metabolism, purine metabolism, vitamin B_6_ metabolism, and phenylalanine-tyrosine-tryptophan biosynthesis as the four most perturbed metabolic pathways in ASD (Fig. 3).

**Fig. 4:**
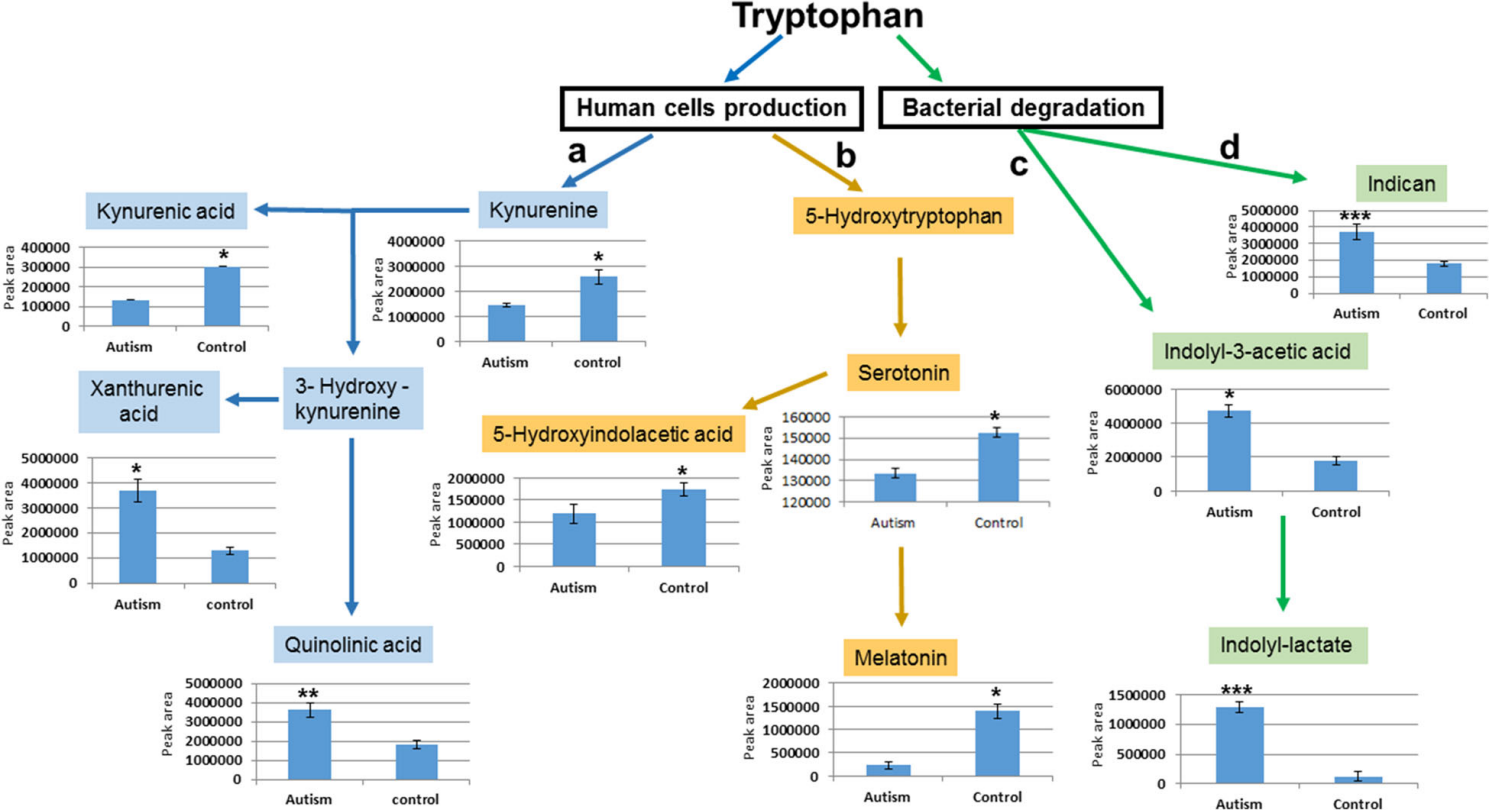
Quantification of tryptophan metabolites: **a** kynurenine pathway; **b** serotonin/melatonin pathway; **c**–**d** bacterial degradation products. *Peak areas* for each metabolite were normalized by urinary specific gravity. Nominal *P* values: *P < 0.05, **P < 0.0.1, ***P < 0.001

Given the relevance of tryptophan-derived compounds in many neural functions, tryptophan metabolism was assessed in greater detail at the level of specific intermediates (Fig. 4):

- The kynurenine pathway displays increases in xanthurenic acid and especially in quinolinic acid, paralleled by a considerable decrease in kynurenic acid (Fig. 4, path A).
- The serotonin pathway shows a significant decrease in melatonin and its catabolite N-acetyl-5-methoxytryptamine, which have the same molecular weight and thus fall under the same MS peak (Fig. 4, path B).
- Bacterial degradation of tryptophan yields in ASD, compared to controls, prominently larger urinary concentrations of indoxyl sulfate and other indole derivatives, including indolyl-3-acetic acid and especially indolyl lactate (Fig. 4, paths C and D).

**Fig. 5:**
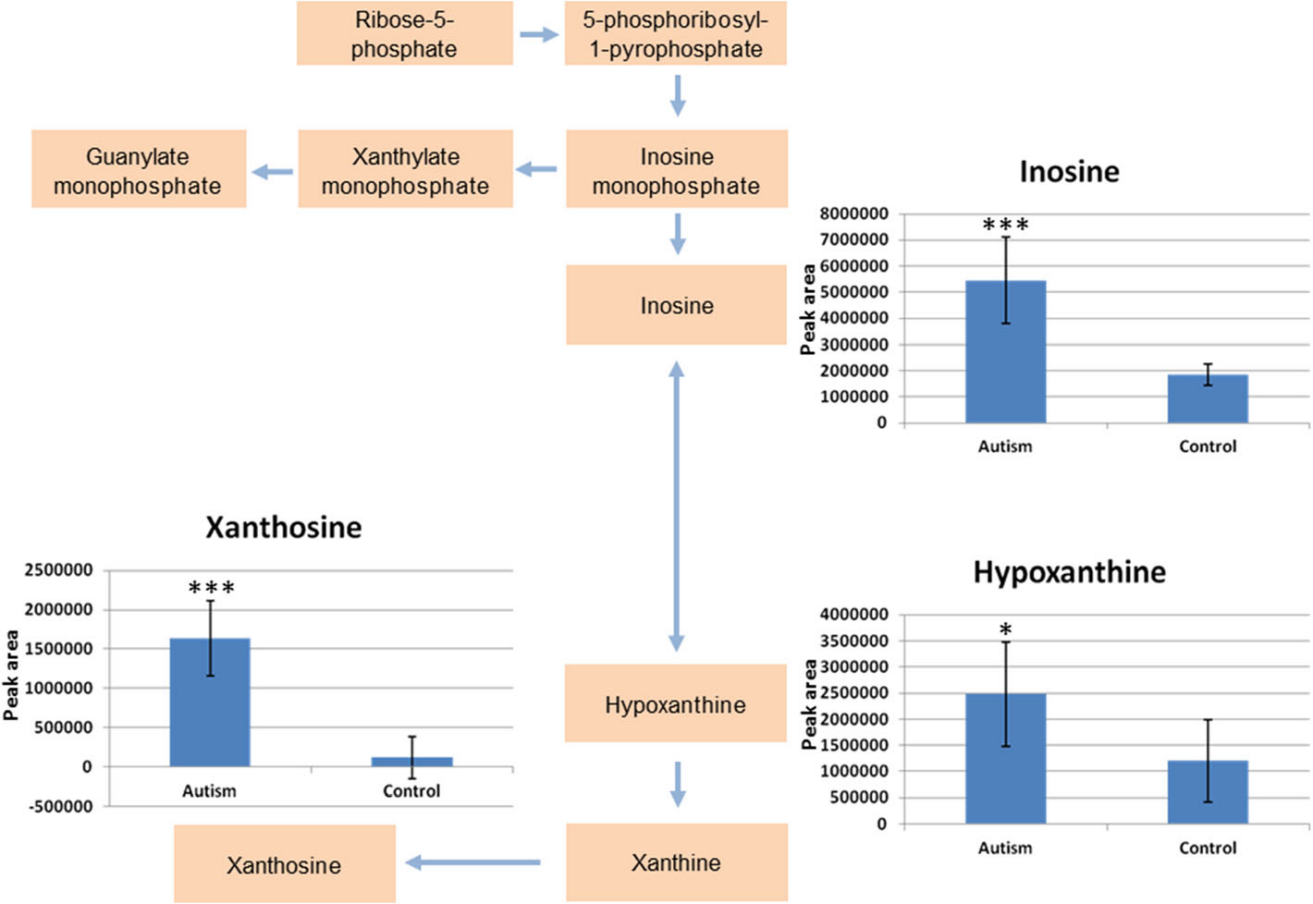
Quantification of purine metabolites. *Peak areas* for each metabolite were normalized by urinary specific gravity. Nominal *P* values: *P < 0.05, **P < 0.0.1, ***P < 0.001

Also, purine metabolism was found to convey sizable discriminative power, because ASD cases display higher urinary concentrations of many purine metabolites compared to controls, including, among others, inosine, hypoxanthine, and xanthosine (Fig. 5).

## Discussion

The present study reports significant urinary metabolomic differences between young children with idiopathic ASD and typically developing controls. At least some of the metabolic perturbations described here may reflect pathophysiologically meaningful abnormalities, possibly bearing functional consequences at the clinical level. Three strengths of the experimental design may have contributed to this positive outcome: (a) a focus on early infancy, by recruiting children within a relatively narrow age window precisely defined on the basis of previous urinary metabolic data [20, 21]; (b) the use of UHPLC-MS paired with HILIC, a very sensitive and reliable method ensuring maximum accuracy in the separation of small urinary solutes [26, 27]; (b) a pathway-centered approach, moving beyond the identification of single urinary ASD markers [10–17], as beautifully exemplified by urinary metabolomic studies of rodent models of ASD [35–37]. In particular, our recruitment strategy substantially differs from previous case-control study designs, minimizing age-dependent heterogeneity by setting data-driven age thresholds (i.e., 2–8 years old) [20, 21], and applying tight age and sex matching between cases and controls. This strategy seemingly circumvents sample size limitations which would apply to an unfocused and unmatched case-control design. Future replications obtained applying similar recruitment criteria will enhance confidence in the pathophysiological relevance and the interethnic generalizability of our findings.

The tryptophan metabolic pathway collectively displays the largest perturbations in ASD (Fig. 3). Over 90–95% of dietary l-tryptophan is usually metabolized along the kynurenine pathway, 1–2% is converted to serotonin, and approximately 4–6% undergoes bacterial degradation prior to gut absorption through the Na^+^-amino acid co-transporter B^0^AT1 (Slc6a19) [38, 39]. The latter pathway yields indole derivatives not produced by mammalian metabolism, such as indoxyl sulfate [40]. Hence, changes in urinary amounts of multiple metabolites provide more reliable evidence of perturbed tryptophan metabolism, as compared to determinations of single metabolites or tryptophan itself, which also suffer from reduced statistical power due to control for multiple testing (Figs. 4 and 5). In the urines of young autistic children, we have indeed observed a substantial increase of xanthurenic acid and especially of quinolinic acid, paralleled by a decrease in kynurenine and kynurenic acid (Fig. 4, path A). This pattern is extremely interesting but must be interpreted with some caution in the absence of parallel assessments of the cerebrospinal fluid (CSF). On the one hand, the enzymes responsible for the synthesis of quinolinic acid and xanthurenic acid are primarily expressed in the microglia and in macrophages, whereas the path leading to kynurenic acid is functional in astrocytes [41]. Hence, it would be tempting to speculate that these opposite trends between cases and controls reflect an abnormal activation of microglia, which has been repeatedly seen in ASD postmortem brains [42–44], even as early as at 4 years of age [45]. On the other hand, urinary levels of quinolinic acid and kynurenic acid reflect peripheral production of these compounds, which do not pass the blood-brain barrier [41]. However, 3-hydroxykynurenine does pass the blood-brain barrier [41]. Interestingly, urinary concentrations of metabolite downstream of this compound (quinolinic acid and xanthurenic acid) are elevated in autistic children, whereas metabolite upstream of 3-hydroxykinurenine (kynurenine and kynurenic acid) are higher among controls (Fig. 4, path A). Conceivably, these trends could reflect an outflow of 3-hydroxykynurenine from the central nervous system (CNS) into the systemic circulation, where macrophage activation presumably at the level of the gut or in other peripheral organs, can transform this compound into quinolinic acid and xanthurenic acid, as well as into nicotinic acid (NAD), in agreement with previous data [10]. It will thus be important to verify this metabolomic scenario in the CSF, because it could have at least two important clinical implications: (a) quinolinic acid acts as gliotoxin, proinflammatory mediator, and pro-oxidant molecule, boosting oxidative stress by stimulating microglia to release large amounts of NO and superoxide; (b) quinolinic acid exerts excitotoxic effects by acting as an *N*-methyl-d-aspartate (NMDA) receptor agonist, stimulating glutamate release, blocking glutamate reuptake into astrocytes, and reducing the activity of glutamine synthase; instead, kynurenic acid exerts neuroprotection via NMDA antagonism at the glycine binding site, as well as antioxidant effects [41, 46, 47]. In summary, the urinary metabolic imbalance documented here, if present also in the CNS, could favor enhanced oxidative stress and the well-known excitation>inhibition imbalance present in ASD, fostering seizures in as many as 20% of autistic individuals [48].

Another consequence of the preferential metabolization of tryptophan along the main branch of the kynurenine pathway is the relative decrease in the production of serotonin and melatonin (Fig. 4, path B). The serotonin pathway sees tryptophan being converted into 5-hydroxytryptophan (5-HTP) by tryptophan hydroxylase and onwards to 5-hydroxytryptamine (5-HT) or serotonin by 5-HTP decarboxylase. Serotonin can then be catabolized to 5-hydroxyindoleacetic acid (5-HIAA) or transformed into N-acetylserotonin by arylalkylamine N-acetyltransferase (AANAT). N-acetylserotonin is further methylated by N-acetylserotonin O-methyltransferase (ASMT) to generate the neurohormone 5-methyl-5-methoxy-N-tryptamine or melatonin. Decreases in the serotonin metabolite 5-HIAA are only modest, while urinary melatonin and its catabolite N-acetyl-5-methoxytryptamine display a more pronounced mean reduction (both share the same molecular weight and fall under the same MS peak, labeled in Fig. 4, path B, as “melatonin” only). This confirms previous assessments performed in plasma or urine [10, 49–52], while lending further support to blunted melatonin synthesis possibly due to reduced ASMT enzyme activity in ASD [53, 54]. Melatonin is synthesized and released by the pineal gland into the systemic circulation and readily passes the blood-brain barrier [55]. Its well-known role in circadian rhythmicity makes it an ideal candidate to explain the frequent occurrence, especially at the onset of ASD and during early infancy, of sleep disorders highly responsive to melatonin as a pharmacological therapy [56].

Metabolites produced by gut bacteria are well-represented also in our ASD sample, as in previous studies [10–17]. In addition to urinary *p*-cresol, found elevated in these same urine samples both here (Fig. 3) and previously using a different technology [20], we also detect a significant increase in indole derivatives of bacterial tryptophan including indolyl 3-acetic acid, indoxyl sulfate, and most prominently, indolyl lactate (Fig. 4, path C). Bacterial species expressing tryptophanase, the enzyme responsible for transforming tryptophan into indole derivatives, include *Escherichia coli*, *Proteus vulgaris*, *Paracolobactrum coliform*, *Achromobacter liquefaciens*, and *Bacteroides* spp. [40]. Once produced in the gut lumen, indole is absorbed, oxidized to indoxyl, conjugated with sulfate, and excreted as urinary indoxyl sulfate. About 3% of tryptophan entered with the diet is excreted as indoxyl sulfate [37]. Additional small amounts of tryptophan are converted into other indole derivatives found elevated here in ASD children, such as indolyl-3-acetic acid and indolyl lactate (Fig. 4, path C). The latter compound and indolyl 3-acetic acid are direct precursors of indolylacrylol glycine, found elevated in ASD by some [57] but not all studies [58, 59]. Predictably, the exact urinary bacterial compounds found elevated in ASD do differ in distinct metabolomic studies. This is not surprising since, in addition to differences in sample demographics and sensitivity of available technologies, ethnicity also exerts profound influences on the microbiome, reflecting dietary, genetic, and immunological specificities involved in the host-microbiome interactions [60]. Despite these discrepancies at the level of single compounds, urinary metabolomic studies consistently report an excess of microbiome-derived urinary metabolites, collectively supporting gut dysbiosis in ASD. These results point toward possible negative effects on CNS function exerted by microbiome-derived metabolites. At least three examples are available, albeit with different degrees of support: (a) urinary *p*-cresol amounts were found correlated with ASD severity [20] or with the intensity of stereotypic behaviors in young autistic children [21]; (b) i.c.v. injection of propionic acid, an enteric-derived short chain fatty acid, produces ASD-like behaviors in the rat [61]; (c) indoxyl sulfate is a known risk factor for cognitive impairment in chronic renal disease [62]: its influx across the blood-brain barrier using the organic anion transporter 3 significantly reduces the efflux of various neurotransmitter metabolites through the same transporter, leading to their accumulation [63]. Importantly, sizable improvements in behavioral and serum metabolome abnormalities were recorded using the maternal immune activation (MIA) rodent model of ASD following the correction of gut dysbiosis using *Bacteroides fragilis* [64].

Purine metabolites are also well represented in the urines of ASD children, which display a large excess of inosine, hypoxanthine, and xanthosine (Figs. 3 and 5). This pattern bears an interesting resemblance to the excess of urinary inosine and hypoxanthine detected in *Fmr1* knock-out mice, an animal model of fragile-X syndrome [35]. Also, mice exposed prenatally to MIA triggered by poly(I:C) injected at E12.5 and E17.5 show an excess of urinary inosine [36]. This excess of urinary purinergic metabolites has been interpreted as part of a “cell danger metabolic response” involving mitochondrial dysfunction, adenosine triphosphate (ATP), and adenosine diphosphate (ADP) release, activation of a variety of purinergic receptors yielding microglial activation, innate, and adaptive immunity responses and leukocyte chemotactics [65]. Inborn errors of purine metabolism are associated with behavioral abnormalities including autistic features [66]. Strikingly, inhibition of purine metabolism by suramin, a competitive antagonist at P2X and P2Y purinergic receptors, reverses behavioral, neurochemical, transcriptional, and metabolomics abnormalities both in the *Fmr1* knock-out mouse and in MIA mice exposed to poly(I:C) during pregnancy [35–37]. Conceivably, this metabolic abnormality, shared between human ASD and genetic/immunological rodent models could thus represent a valuable biomarker to help guide therapeutic interventions. In addition, the cell danger response also yields relative vitamin B_6_ deficiency and the enzyme kynureninase is B_6_ dependent [65]; hence, a cell danger metabolic response in the presence of adequate tryptophan intake could also explain the decreased kynurenine and increased xanthurenic and quinolinic acid observed here (Fig. 4). Interestingly, these abnormalities have been sometimes overcome with vitamin B_6_ supplementation [67], a therapeutic approach initially proposed for ASD in conjunction with magnesium supplementation [68]. In light of the present data, B_6_-Mg^++^ supplementation in ASD may deserve further scrutiny in urinary biomarker-driven therapeutic trials, as no firm conclusion on its potential efficacy has yet been reached [69].

## Conclusions

Targeting young autistic children and tightly matched controls, using the sensitive approach HILIC UHPLC-MS, and applying metabolic pathway analysis, we identified several urinary metabolic pathways significantly altered in ASD: tryptophan, purine, and vitamin B_6_ metabolisms; phenylalanine, and tyrosine biosynthesis; and to a lesser extent, pantothenate and CoA, riboflavin, and pyrimidine metabolisms. Several of these same pathways, especially tryptophan, purine, and gut microbiome metabolisms, are also abnormal in animal models of ASD and provide very interesting leads toward possible pathophysiological explanations for specific symptoms present in many autistic children, such as seizures and sleep disorders. These metabolic abnormalities may apply to young children only, as suggested by studies of urinary *p*-cresol [20, 21]. It will indeed be very important to now perform a similar metabolomic assessment on ASD individuals and controls older than 8 years of age. Investigations of CSF metabolomics will be necessary to verify to what extent peripheral results reflect CNS pathophysiology. Finally, studies involving other diagnostic groups bordering with ASD, such as ADHD, intellectual disability, expressive language disorder, and obsessive-compulsive disorder, will be required to assess the disease specificity of the metabolomic abnormalities reported here and to determine their potential value as ASD-specific biomarkers, possibly able to aid clinicians in providing more reliable diagnoses in early infancy.

## Additional files

Additional file 1: Table S1. Demographic and clinical characteristics of the autistic sample (*N* = 30, unless otherwise specified). Typically developing controls were tightly sex- and age-matched, with M:F = 22–8, age 5.03 ± 0.32 years, and no clinical evidence of ASD-related DSM-IV diagnoses or intellectual disability. (DOCX 21 kb)
Additional file 2: Supplementary methods and references. (DOCX 15 kb)
Additional file 3: Figure S1. Accurate mass and MS/MS fragmentation data for (a) kynurenine, (b) melatonin, and (c) tryptophan. (TIF 191 kb)
Additional file 4: Detailed and summary statistics for metabolites displayed in Figs. 4 and 5. (DOCX 17 kb)
Additional file 5: Summary statistics. (XLSX 10 kb)
Additional file 6: Figure S2. Q2 and R2 data pertaining to the PCA. **p* < 0.05 refers to the best values of the currently selected measures (Q2). (TIF 233 kb)
Additional file 7: Figure S3. ROC curve for the top 25 most discriminating metabolites between ASD cases and controls, displayed in Fig. 2. (TIF 131 kb)

## Abbreviations

5-HIAA: 5-hydroxyindoleacetic acid
5-HT: 5-hydroxytryptamine
5-HTTP: 5-hydroxytryptophan
AANAT: N-acetylserotonin by arylalkylamine N-acetyltransferase
ADHD: attention deficit hyperactivity disorder
ADP: adenosine diphosphate
ASD: autism spectrum disorder
ASMT: N-acetylserotonin O-methyltransferase
ATP: adenosine triphosphate
CNS: central nervous system
CSF: cerebrospinal fluid
ESI: electrospray ionization
GC: gas chromatography
HILIC: hydrophilic interaction chromatography
KEGG: Kyoto Encyclopedia of Genes and Genomes
LC: liquid chromatography
MetPA: metabolic pathway analysis
MIA: maternal immune activation
MS: mass spectroscopy
NAD: nicotinic acid
NMDA: *N*-methyl-d-aspartate
NMR: nuclear magnetic resonance
OPLS-DA: orthogonal partial least squares discriminant analysis
TOF: time of flight
VIP: variable influence on the projection
