# Supplementary material for: Urinary metabolomics of young Italian autistic children supports abnormal tryptophan and purine metabolism
Source: Mol Autism. 2016 Nov 24;7:47. doi: 10.1186/s13229-016-0109-5 (PMC5121959; doi:10.1186/s13229-016-0109-5)
Supplement: Additional file 1: Table S1. — Demographic and clinical characteristics of the autistic sample (N = 30, unless otherwise specified). Typically developing controls were tightly sex- and age-matched, with M:F = 22–8, age 5.03 ± 0.32 years, and no clinical evidence of ASD-related DSM-IV diagnoses or intellectual disability. (DOCX 21 kb) [file 13229_2016_109_MOESM1_ESM.docx]

**Additional file 1: Table S1.** Demographic and clinical characteristics of the autistic sample.

|  | | | **N** | **Mean/Median** | **Range** |
| --- | --- | --- | --- | --- | --- |
| *Age in yrs (mean± S.E.M.):* | | | N=30 | 4.83 ± 0.30 | 2-7 |
| *Median ADOS scores:* | | | N=19 |  |  |
| *1) Language and communication* | | |  | 4.90 | 0-10 |
| *2) Social interactions* | | |  | 8.80 | 4-13 |
| *3) Play and imagination* | | |  | 3.30 | 1-5 |
| *4) Stereotypies* | | |  | 3.10 | 0-6 |
| *5) Abnormal behaviors* | | |  | 0.30 | 0-2 |
| *Median ADI scores:* | | | N=7 |  |  |
| *A) Reciprocal social interactions* | | |  | 18.50 | 5-30 |
| *B) Language/Communication* | | |  | 13.80 | 9-23 |
| *C) Restricted, repetitive and stereotyped behaviors and interests* | | |  | 5.20 | 2-9 |
| *D) Behavioral abnormalities at or prior to 36 months of age* | | |  | 4.30 | 3-5 |
| *Median VABS scores:* | | | N=8 |  |  |
| *Communication* | | |  | 77.0 | 51-113 |
| *Daily living skills* | | |  | 72.5 | 20-112 |
| *Socialization* | | |  | 75.10 | 33-114 |
| *Motor skills* | | |  | 97.60 | 63-120 |
| *Composite* | | |  | 79.60 | 48-117 |
|  | | | **N** | **Percent** | |
| *Gender:* | | *Male* | 22 | 73.3% | |
|  | | *Female* | 8 | 26.7% | |
|  | | *M/F ratio* | 2.8:1 |  | |
| *Family type:* | | *Simplex* | 27 | 90.0% | |
|  | | *Multiplex* | 3 | 10.0% | |
| *DSM-IV Diagnosis:* | | *Autistic Disorder* | 18 | 60.0% | |
|  | | *Asperger Syndrome* | 0 | 0.0% | |
|  | | *PDD-NOS* | 12 | 40.0% | |
| *I.Q. (N=23):* | *>70* | | 8 | 34.8% | |
|  | *< 70* | | 15 | 65.2% | |
