# Supplementary material for: Urinary metabolomics of young Italian autistic children supports abnormal tryptophan and purine metabolism
Source: Mol Autism. 2016 Nov 24;7:47. doi: 10.1186/s13229-016-0109-5 (PMC5121959; doi:10.1186/s13229-016-0109-5)
Supplement: Additional file 2: — Supplementary methods and references. (DOCX 15 kb) [file 13229_2016_109_MOESM2_ESM.docx]

**Additional file 2: Supplementary Methods and References**

*Clinical characterization*

Children fulfilling DSM-IV diagnostic criteria for Autistic Disorder, Asperger Disorder, or PDDNOS^1^ were screened for non-syndromic autism using MRI, EEG, audiometry, urinary aminoacid and organic acid measurements, cytogenetic and fragile-X testing. Patients with dysmorphic features were excluded even in the absence of detectable cytogenetic alterations. Patients with sporadic seizures (i.e., < 1 every 6 months) were included; patients with frequent seizures or focal neurological deficits were excluded. Autistic behaviors were assessed using the Autism Diagnostic Observation Schedule (ADOS),^2^ and the Autism Diagnostic Interview - Revised (ADI-R),^3^ as well as the Children Autism Rating Scales (CARS);^4^ adaptive functioning was assessed using the Vineland Adaptive Behavior Scales (VABS);^5^ I.Q. was determined using either the Griffith Mental Developmental Scales, the Coloured Raven Matrices, the Bayley Developmental Scales or the Leiter International Performance Scale. Other clinical characteristics and comorbidites were assessed using a previously published questionnaire.^6^

*References:*

1. American Psychiatric Association. Diagnostic and Statistical Manual of Mental Disorders, 4th edn. American Psychiatric Association: Washington DC, 1994.
2. Lord C, Rutter M, DiLavore PC, Risi S. (2002). *ADOS, Autism Diagnostic Observation Schedule.* Los Angeles, CA: Western Psychological Services [Italian version by Tancredi R, Saccani M, Persico AM, Parrini B, Igliozzi R, Faggioli R, editors (2005). Florence, Italy: Organizzazioni Speciali].
3. Rutter M, Le Couter A, Lord C. (2003). *ADI-R, Autism Diagnostic Interview - Revised.* Los Angeles, CA: Western Psychological Services [Italian version by Faggioli R, Saccani M, Persico AM, Tancredi R, Parrini B, Igliozzi R, editors (2005). Florence, Italy: Organizzazioni Speciali].
4. Schopler E, Reichler RJ, Rochen Renner BR. *The Childhood Autism Rating Scale for diagnostic screening and classification of autism.* Irvington: New York, 1986.
5. Sparrow SS, Balla DA, Cicchetti DV. *Vineland Adaptive Behavior Scales - Survey Form*. American Guidance Service Inc: Circle Pines, MN, 1984.
6. Sacco R, Curatolo P, Manzi B, Militerni R, Bravaccio C, Frolli A, Lenti C, Saccani M, Elia M, Reichelt KL, Pascucci T, Puglisi-Allegra S, Persico AM. Principal pathogenetic components and biological endophenotypes in autism spectrum disorders. Autism Res. 2010; 3:237-252.
