# Supplementary figures and images for: Urinary metabolomics of young Italian autistic children supports abnormal tryptophan and purine metabolism
Source: Mol Autism. 2016 Nov 24;7:47. doi: 10.1186/s13229-016-0109-5 (PMC5121959; doi:10.1186/s13229-016-0109-5)

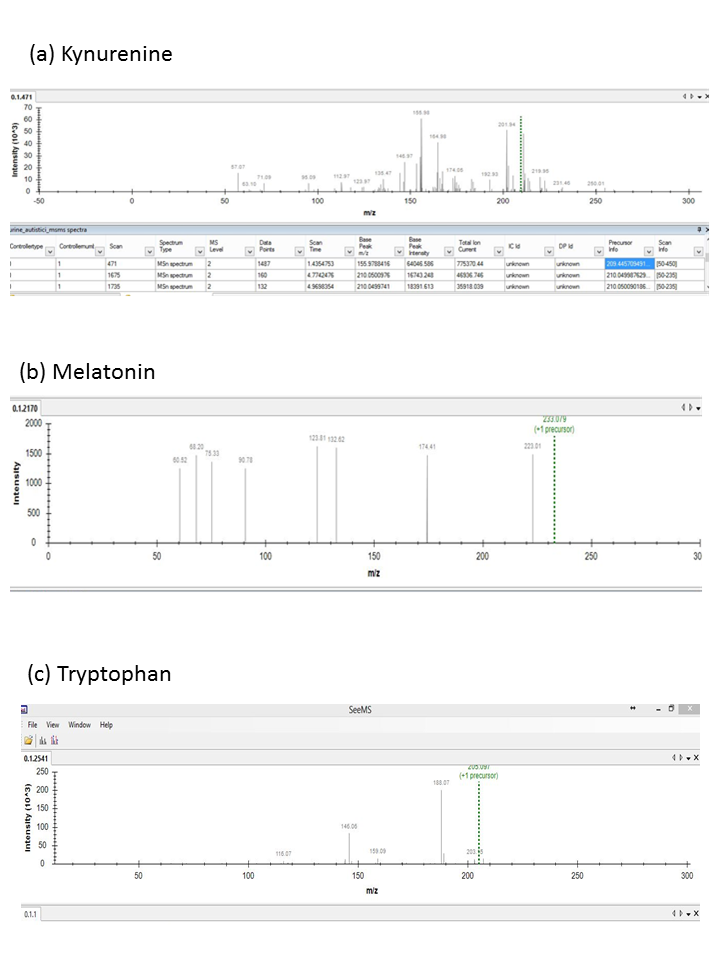

Supplement: Additional file 3: Figure S1. — Accurate mass and MS/MS fragmentation data for (a) kynurenine, (b) melatonin, and (c) tryptophan. (TIF 191 kb) [file 13229_2016_109_MOESM3_ESM.tif]

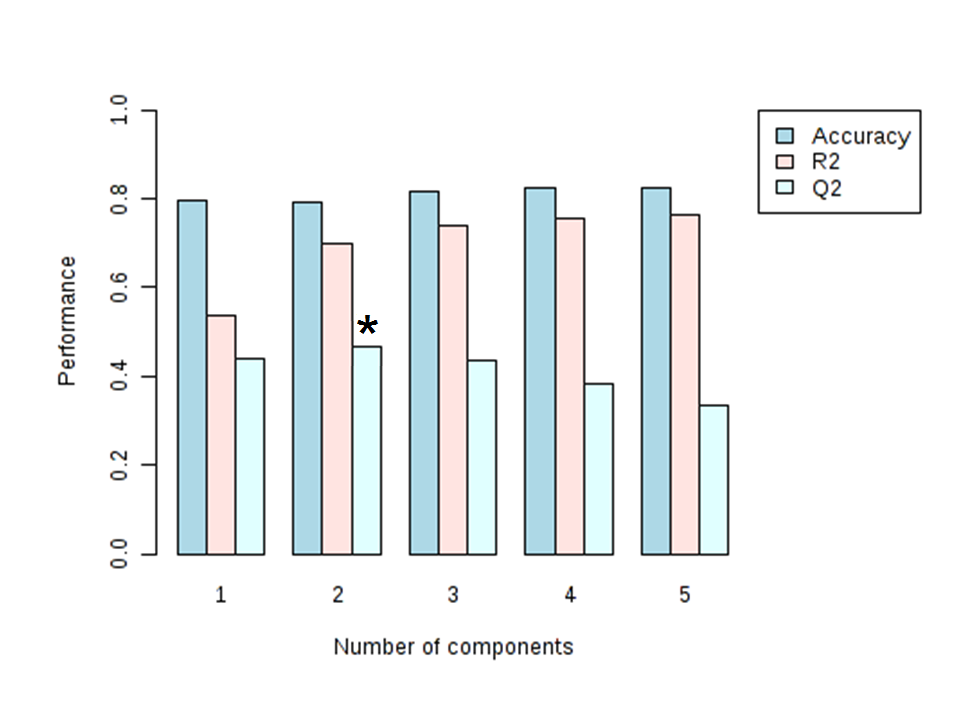

Supplement: Additional file 6: Figure S2. — Q2 and R2 data pertaining to the PCA. *p < 0.05 refers to the best values of the currently selected measures (Q2). (TIF 233 kb) [file 13229_2016_109_MOESM6_ESM.tif]

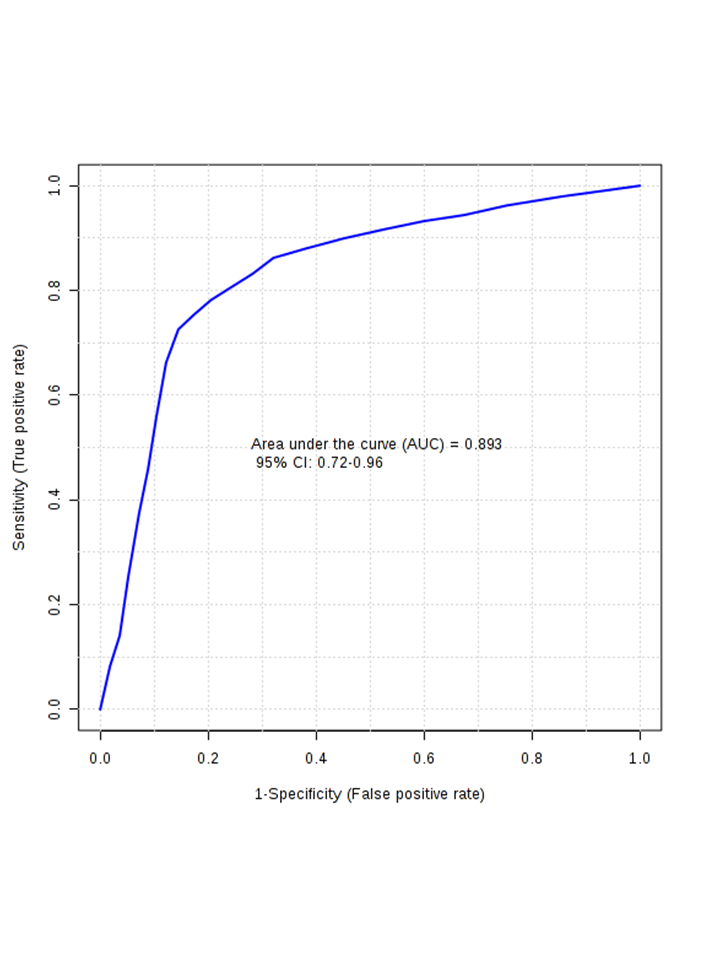

Supplement: Additional file 7: Figure S3. — ROC curve for the top 25 most discriminating metabolites between ASD cases and controls, displayed in Fig. 2. (TIF 131 kb) [file 13229_2016_109_MOESM7_ESM.tif]
