# Supplementary material for: Urinary metabolomics of young Italian autistic children supports abnormal tryptophan and purine metabolism
Source: Mol Autism. 2016 Nov 24;7:47. doi: 10.1186/s13229-016-0109-5 (PMC5121959; doi:10.1186/s13229-016-0109-5)
Supplement: Additional file 4: — Detailed and summary statistics for metabolites displayed in Figs. 4 and 5. (DOCX 17 kb) [file 13229_2016_109_MOESM4_ESM.docx]

**Statistics for Figure 4**

**Kynurenine**

mean1 eq: 1061091 (variance= 561879616) (se= 4327.7385)

mean2 eq: 2707808 (variance= 297286564) (se= 3147.9441)

Probability that var1<var2

p=0.04595 (left: 0.954; double: 0.092)

Difference between means:

M1-M2=1061091-2707808=-1646717.1

sd=38951; se=5351.5

95% CI of difference:

-1657205.9254 <-1646717.1< -1636228.2746 (Wald)

t-difference: -307.71

df-t: 52.5; p= 0

(left p: 1; two sided: 0)

**Quinolinic Acid**

mean1 eq: 3424397 (variance= 1466890000) (se= 6992.5913)

mean2 eq: 1907661 (variance= 585446416) (se= 4417.565)

Probability that var1<var2

p=0.00795 (left: 0.992; double: 0.016)

Difference between means:

M1-M2=3424397-1907661=1516736

sd=57879; se=8271.1

95% CI of difference:

1500525 <1516736< 1532947 (Wald)

t-difference: 183.378

df-t: 48.5; p= 1

(left p: 0; two sided: 0)

**Xanthurenic acid**

mean1 eq: 3698260 (variance= 2089588782681) (se= 263918.4712)

mean2 eq: 1303507 (variance= 1083995404801) (se= 190087.2962)

Probability that var1<var2

p=0.04121 (left: 0.9588; double: 0.0824)

Difference between means:

M1-M2=3698260-1303507=2394753

sd=2361308; se=325248

95% CI of difference:

1757278 <2394753< 3032228 (Wald)

t-difference: 7.363

df-t: 52.2; p= 1

(left p: 0; two sided: 0)

**Melatonin**

mean1 eq: 227080 (variance= 1466821343376) (se= 221119.9782)

mean2 eq: 1395959 (variance= 730589015025) (se= 156054.3725)

Probability that var1<var2

p=0.0327 (left: 0.9673; double: 0.0654)

Difference between means:

M1-M2=227080-1395959=-1168879

sd=1954370; se=270642

95% CI of difference:

-1699328.2643 <-1168879< -638429.7357 (Wald)

t-difference: -4.319

df-t: 51.6; p= 4.0E-5

(left p: 1; two sided: 0)

**Indolyl lactate**

mean1 eq: 1288788 (variance= 41312188516) (se= 37108.9336)

mean2 eq: 126673 (variance= 12428905225) (se= 20354.2831)

Probability that var1<var2

p=0.00091 (left: 0.9991; double: 0.0018)

Difference between means:

M1-M2=1288788-126673=1162115

sd=283926; se=42325

95% CI of difference:

1079160 <1162115< 1245070 (Wald)

t-difference: 27.457

df-t: 44.5; p= 1

(left p: 0; two sided: 0)

**Indican**

mean1 eq: 3892860 (variance= 9575405316) (se= 17865.6144)

mean2 eq: 1503507 (variance= 2892826225) (se= 9819.7526)

Probability that var1<var2

p=0.00094 (left: 0.9991; double: 0.0018)

Difference between means:

M1-M2=3892860-1503507=2389353

sd=136843; se=20386

95% CI of difference:

2349396 <2389353< 2429310 (Wald)

t-difference: 117.203

df-t: 44.6; p= 1

(left p: 0; two sided: 0)

**indolyl 3 acetic acid**

mean1 eq: 4753109 (variance= 125872396225) (se= 64774.5825)

mean2 eq: 1768662 (variance= 58343987025) (se= 44099.8817)

Probability that var1<var2

p=0.02126 (left: 0.9787; double: 0.0426)

Difference between means:

M1-M2=4753109-1768662=2984447

sd=560324; se=78362

95% CI of difference:

2830861 <2984447< 3138033 (Wald)

t-difference: 38.086

df-t: 50.6; p= 1

(left p: 0; two sided: 0)

**5-hydroxy-indolacetic acid**

z for 95% CI= 1.96

declare p larger than alpha=0.05 not significant.

mean1 eq: 1185880 (variance= 75874906116) (se= 50290.7898)

mean2 eq: 1743152 (variance= 37925225536) (se= 35555.2272)

Probability that var1<var2

p=0.03338 (left: 0.9666; double: 0.0668)

Difference between means:

M1-M2=1185880-1743152=-557272

sd=444967; se=61590

95% CI of difference:

-677986.5584 <-557272< -436557.4416 (Wald)

t-difference: -9.048

df-t: 51.7; p= 0

(left p: 1; two sided: 0)

**Serotonin**

Mean 1: 132545

Mean 2: 157845

N1: 30

N2: 30

Std Dev.1: 58784

Std Dev.2: 41452

mean1 eq: 132545 (variance= 3455558656) (se= 10732.4409)

mean2 eq: 157845 (variance= 1718268304) (se= 7568.0652)

Probability that var1<var2

p=0.03239 (left: 0.9676; double: 0.0648)

Difference between means:

M1-M2=132545-157845=-25300

sd=94811; se=13132

95% CI of difference:

-51039.151 <-25300< 439.15 (Wald)

t-difference: -1.927

df-t: 51.6; p= 0.0299

(left p: 0.9701; two sided: 0.0598)

**kynurenic acid**

mean1 eq: 132300 (variance= 370370025) (se= 3513.6402)

mean2 eq: 303009 (variance= 183467025) (se= 2472.9673)

Probability that var1<var2

p=0.03168 (left: 0.9683; double: 0.0634)

Difference between means:

M1-M2=132300-303009=-170709

sd=31004; se=4296.7

95% CI of difference:

-179130.3025 <-170709< -162287.6975 (Wald)

t-difference: -39.731

df-t: 51.6; p= 0

(left p: 1; two sided: 0)

**Statistics for Figure 5:**

**Xanthosine**

mean1 eq: 1634879 (variance= 224642208009.5) (se= 86533.6559)

mean2 eq: 0.0115 (sd= 245018.648) (se= 44734.0801)

Probability that var1<var2

p=0.00033 (left: 0.9997; double: 0.0006)

Difference between means:

M1-M2=1634879-0.0115=1634879

sd=642236; se=97413

95% CI of difference:

1443953 <1634879< 1825804 (Wald)

t-difference: 16.783

df-t: 43; p= 1

(left p: 0; two sided: 0)

**Inosine**

mean1 eq: 5456060 (variance= 1806344064009) (se= 245380.2535)

mean2 eq: 1862601 (variance= 523832880169) (se= 132140.4405)

Probability that var1<var2

p=0.00067 (left: 0.9993; double: 0.0014)

Difference between means:

M1-M2=5456060-1862601=3593459

sd=1859461; se=278698

95% CI of difference:

3047220 <3593459< 4139698 (Wald)

t-difference: 12.894

df-t: 44; p= 1

(left p: 0; two sided: 0)

**Hypoxantine**

mean1 eq: 2479646 (variance= 1004965424604.6) (se= 183026.9037)

mean2 eq: 1196642 (variance= 527783562115.85) (se= 132637.7978)

Probability that var1<var2

p=0.04412 (left: 0.9559; double: 0.0882)

Difference between means:

M1-M2=2479646-1196642=1283004

sd=1643618; se=226035

95% CI of difference:

839983 <1283004< 1726024 (Wald)

t-difference: 5.676

df-t: 52.4; p= 1

(left p: 0; two sided: 0)
